# Ferroptosis and cuproptosis in periodontitis: recent biological insights and therapeutic advances

**DOI:** 10.3389/fimmu.2025.1526961

**Published:** 2025-02-24

**Authors:** Tengyi Zheng, Fumiao Lu, Peihang Wu, Yangan Chen, Rongxin Zhang, Xin Li

**Affiliations:** ^1^ Department of Pharmacy, Guangdong Pharmaceutical University, Guangzhou, China; ^2^ School of Life Sciences and Biopharmaceutics, Guangdong Pharmaceutical University, Guangzhou, China; ^3^ Department of Endodontics, Southern Medical University Stomatological Hospital, Guangzhou, China

**Keywords:** periodontitis, ferroptosis, cuproptosis, oxidative stress, glutathione, autophagy

## Abstract

Periodontitis is a significant global public health issue associated with the onset and progression of various systemic diseases, thereby requiring additional research and clinical attention. Although ferroptosis and cuproptosis have emerged as significant areas of research in the medical field, their precise roles in the pathogenesis of periodontitis remain unclear. We aim to systematically summarize the current research on ferroptosis and cuproptosis in periodontal disease and investigate the roles of glutathione pathway and autophagy pathway in connecting ferroptosis and cuproptosis during periodontitis. Further, we propose that a homeostatic imbalance of copper and iron, driven by periodontal pathogens, may contribute to elevated periodontal oxidative stress, representing a potential unifying link between ferroptosis and cuproptosis involved in periodontitis. This article presents a comprehensive overview of the molecular mechanisms underlying ferroptosis and cuproptosis in periodontitis, offering novel theoretical insights into its pathogenesis and potential therapeutic targets.

## 1 Introduction

Periodontitis is a multifaceted inflammatory oral disease initiated by pathogenic biofilms. This condition might lead to persistent destruction of the periodontium, characterized by periodontal inflammation and alveolar bone loss, and subsequently tooth loss. Periodontitis represents a significant global health threat, affecting individuals across all age groups and contributing to a considerable public health burden (1). Numerous studies have confirmed that periodontitis is not only limited to a significant oral health concern but also closely associated with the initiation and development of systemic or organ-specific diseases (2–8). However, the underlying molecular mechanisms of periodontitis remain poorly understood. Therefore, investigating its pathogenesis and uncovering novel therapeutic targets are pivotal for advancing safe and efficacious treatments for periodontitis, with profound clinical implications for oral and systemic health.

Recently, the concept of cell death has evolved to primarily encompass two categories, including necrosis and programmed cell death (PCD). PCD is a series of regulated cellular suicide mechanisms to maintain organismal homeostasis, including apoptosis, autophagy, necroptosis, pyroptosis (9–12). Several novel PCD pathways, such as ferroptosis and cuproptosis, have gradually become recent research hotspots in the medical field (13, 14). Although plenty of studies have found that PCD may be involved in the periodontal inflammatory response, the precise roles of ferroptosis and cuproptosis in the pathogenic mechanism of periodontitis have not yet been fully clarified. Therefore, the present article aims to systematically synthesize empirical findings that elucidate the critical function of ferroptosis and cuproptosis during periodontitis and investigate the roles of glutathione (GSH) pathway and autophagy pathway in linking ferroptosis and cuproptosis within periodontal inflammation. Furthermore, we suppose that a homeostatic imbalance of copper and iron, mediated by periodontal pathogens, may constitute a significant factor contributing to elevated periodontal oxidative stress, potentially representing a unifying mechanism that elucidates the intricate interconnection between the two forms of PCD during periodontitis. A better understanding of these pathogenic mechanisms may guide the development of innovative therapeutic approaches for periodontitis.

## 2 Ferroptosis in periodontitis

Ferroptosis, a novel form of PCD characterized by an iron-dependent oxidative imbalance in the intracellular microenvironment, is primarily triggered by the dysfunction of glutathione peroxidase 4 (GPX4), an important regulator of intracellular redox homeostasis (13). Iron balance serves a vital function in the redox cycle reactions to carry out normal biological functions, while intracellular iron overload can facilitate the generation of substantial quantities of reactive oxygen species (ROS) and lipid peroxidation through the Fenton reaction, leading to exaggerated inflammatory cascades that underlie the pathogenesis of inflammatory diseases (15).

Research has increasingly focused on the role of ferroptosis in periodontal inflammation. Extensive bioinformatics analyses have indicated that ferroptosis is involved in the etiology and progression of periodontitis (16–23). Both basic and clinical research has also unveiled common pathophysiological features of periodontitis and ferroptosis, such as oxidative stress and lipid peroxidation (24–27). Elevated iron levels are closely associated with the initiation and severity of periodontitis, indicating that alterations in iron metabolism may occur during periodontitis and thus provide possible positive feedback that reinforces periodontal damage (28–31). An excessive level of iron can exacerbate ROS generation within periodontal tissues and accelerate susceptibility to infection by periodontal pathogens, ultimately inducing cellular ferroptosis and subsequent periodontium damage (31–36). Therefore, iron-dependent oxidative stress likely underlies both periodontitis and ferroptosis. Nevertheless, the specific mechanisms by which ferroptosis contributes to periodontitis remain incompletely elucidated.

Recently, increasing evidence has revealed an association between periodontitis and ferroptosis, indicating that ferroptosis may be a crucial risk factor for the development and progression of periodontal inflammation. Butyrate, a short-chain fatty acid from periodontal pathogens, promoted nuclear receptor coactivator 4 (NCOA4)-mediated ferritinophagy and ferroptosis in periodontal ligament fibroblasts through p38/hypoxia inducible factor-1α (HIF-1α) signaling and bromodomain-containing protein 4 (BRD4)/cyclin-dependent kinase 9 (CDK9) complexes (37). Similarly, lncRNA LINC00616 aggravated ferroptosis in human periodontal ligament stem cells (hPDLSCs) via the microRNA-370/transferrin receptor axis (38). Activating transcription factor 3 (AFT3) inhibited osteogenic differentiation of lipopolysaccharide (LPS)-stimulated hPDLSCs by activating ferroptosis through the nuclear factor erythroid 2-related factor 2 (NRF2)/heme oxygenase 1 (HO-1) pathway (39). Other empirical studies have also found that ferroptosis acts as a catalyst in the progression of periodontitis in human gingival fibroblasts (HGFs) and murine models (40, 41). Moreover, IL-17 administration alleviated osteoblast ferroptosis and promoted osteogenic differentiation via the direct interaction of phosphorylated signal transducer and activator of transcription 3 (STAT3) with NRF2 in periodontitis models (42). Inhibition of zinc finger DHHC-type palmitoyl transferases 16 (ZDHHC16) facilitated osteogenic differentiation of dental pulp stem cells by inhibiting ferroptosis through cAMP-response element binding protein (CREB) pathway, suggesting a negative association between ferroptosis and alveolar bone repair (43). These findings suggest that ferroptosis may be a risk factor for periodontitis development. Appropriate inhibition of ferroptosis may have certain clinical application value in periodontitis treatment (**Table 1**) (44–50).

**Table 1:** Summary of periodontitis treatment based on ferroptosis inhibitors. .

Ferroptosis inhibitors	Research model	Results	Reference
Ferrostatin-1	Rats	Ferrostatin-1 induced significant upregulation of SLC3A2/SLC7A11 and GPX4 while concomitantly suppressing inflammatory cytokine release.	(44)
Curcumin	Mice	Curcumin and resveratrol played a protective role against ferroptosis through the SLC7A11/GPX4 axis.	(45)
Resveratrol			(46)
Bomidin	Mice; hPDLSCs	Bomidin mitigated ferroptosis by activating the KEAP1/NRF2 pathway, ultimately alleviating the inflammatory response in periodontitis therapy.	(47)
Osteoimmuno-modulatory biopatch	Rats; hPDLSCs	Osteoimmunomodulatory biopatch improved osteogenic differentiation of hPDLSCs and inhibited periodontitis by simultaneously regulating IL-17 and ferroptosis.	(48)
Peroxiredoxin 6	HGFs	Peroxiredoxin 6 alleviated LPS-induced inflammation and ferroptosis in periodontitis via regulating NRF2 signaling.	(49)
ALDH2	Rats; hPDLSCs	ALDH2 facilitated osteogenic differentiation of hPDLSCs and reduced periodontal inflammation by blocking ferroptosis via activating NRF2 pathway.	(50)

*SLC3A2*, Solute carrier family 3 member 2; *SLC7A11*, Solute carrier family 7, member 11; *GPX4*, Glutathione peroxidase 4; *hPDLSCs*, Human periodontal ligament stem cells; *KEAP1*, Kelch-1ike ECH-associated protein l; *NRF2*, Erythroid 2-related factor 2; *IL-17*, Interleukin-17; *HGF*, Human gingival fibroblast; *LPS*, Lipopolysaccharide; *ALDH2*, Aldehyde dehydrogenase 2.

Significantly, periodontitis has also been demonstrated to be a potential risk factor for systemic diseases through activating ferroptosis in other organs. The association is largely attributed to the strong epidemiological link between periodontal pathogens and systemic disorders, mediated by the ability of oral microbiota to ectopically colonize distant tissues and induce systemic inflammation through aberrant secretion of proinflammatory cytokines. Xiong et al. underscored the critical roles of periodontal pathogenic bacteria in driving ferroptosis in lung tissue, aggravating chronic obstructive pulmonary disease (51). *Porphyromonas gingivalis* (*P.g*) could exacerbate alcoholic liver disease and nonalcoholic fatty liver disease in murine models, potentially mediated by ferroptosis activation in hepatocytes (52, 53). Additionally, indoxyl sulfate in the gingival crevicular fluid from mice with chronic kidney disease promoted ferroptosis-mediated osteogenic differentiation disorder in MC3T3-E1 cells via blocking the SLC7A11/GPX4 pathway (54).

In summary, ferroptosis is regarded a potential periodontal risk factor, associated with the incidence and severity of periodontitis and related systemic disease. A positive feedback loop may exist between periodontal pathogens and iron concentration in periodontitis. Periodontal pathogens induce iron overload to disrupt the antioxidant system in the periodontium by degrading iron-binding proteins, while elevated iron concentrations may further increase the susceptibility of periodontal tissue to infection and oxidative stress (28, 31, 34, 35, 55, 56). However, previous research has shown that Saikosaponin A attenuate alveolar bone resorption in experimental periodontitis rat models by promoting ferroptosis of osteoclasts via the NRF2/SLC7A11/GPX4 axis, indicating a protective role of ferroptosis against alveolar bone loss (57). Ferroptosis may exhibit different regulatory roles in periodontitis depending on the types of periodontal cells in which ferroptosis occurs. Notably, ferroptosis inhibition as a therapeutic approach for periodontitis is in the exploratory data analysis phase and warrants further study. Given the presence of similar characteristic markers (*e.g.*, iron and ROS) involved in autophagy, apoptosis and pyroptosis, it remains unclear whether ferroptosis coexists with other types of PCD during periodontitis and their specific correlation in periodontal disease. Differences between animal models and humans have generally led to the perception that interventions of ferroptosis inhibitors on periodontitis in animals may not be reproducible in human. Besides, the intricate regulatory networks underlying ferroptosis remain incompletely characterized, which may limit the therapeutic efficacy of single-target inhibitors across diverse pathological conditions. Current pharmacological interventions targeting ferroptosis inhibition are hampered by insufficient tissue selectivity and nonspecific biodistribution, resulting in ineffective targeting of periodontal lesional microenvironments.

## 3 Cuproptosis in periodontitis

Copper is an essential trace metal for human health, acting as a catalytic cofactor in various enzyme-driven physiological processes. Cuproptosis is a recently discovered form of PCD triggered by excessive copper, which is highly correlated with mitochondrial respiration and protein lipoylation. Copper overload stimulates lipoylated protein aggregation and subsequent iron−sulfur cluster protein loss through direct binding to lipoylated components in the tricarboxylic acid (TCA) cycle, resulting in proteotoxic stress and subsequent cell death (14, 58–60). The discovery of cuproptosis may provide new insights into molecular mechanisms and therapeutic targets for various pathological conditions.

Growing evidence indicates potential crosstalk between cuproptosis and periodontitis. Significantly elevated copper levels in saliva and serum of periodontitis patients compared to healthy controls indicated disrupted copper homeostasis and a positive correlation with the periodontal index (61, 62). In addition, 11 cuproptosis-related hub genes associated with periodontitis were identified by bioinformatics analysis and further verified in periodontal tissue from periodontitis patients and healthy controls via real-time quantitative PCR and immunohistochemistry (63). Liu et al. also revealed that the intracellular copper concentration tripled in *P.g* LPS-treated HGFs compared to controls (64). Furthermore, age-associated differences in cuproptosis-related gene expression were noted in gingival tissue biopsies from experimental periodontitis macaque models (65). In contrast, copper chelator tetrathiomolybdate significantly reduced cuproptosis-associated marker levels in LPS-stimulated RAW 264.7 cells and murine periodontitis models, suggesting that cuproptosis inhibition could alleviate macrophage dysfunction in periodontitis (66). Similarly, curcumin gel decreased copper levels, proinflammatory cytokines, and clinical indicators in periodontitis patients (67).

Cuproptosis is considered a possible PCD contributing to periodontal inflammation, offering more comprehensive understanding of periodontitis pathogenesis. Dual immunomodulatory effect of copper complicates the interaction between pathogens and host (68, 69). Invading microorganisms must acquire copper from host to ensure an adequate supply of essential cuproenzymes during infection, whereas excessive copper has a substantial bactericidal effect through ROS production and enzyme mismetalation. Hence, we hypothesize that the host immunomodulation response to periodontal infection impacts copper homeostasis in the periodontium, leading to cuproptosis and initiation of periodontitis. Changes in the inflammatory microenvironment likely intensify copper-induced cytotoxicity and oxidative damage to periodontal tissue by further raising copper levels (70, 71). However, the characteristic markers of cuproptosis in periodontal tissues and its precise relationship with other types of PCD in periodontitis pathogenesis are ambiguous. In addition, further research is warranted to elucidate whether cuproptosis serves as a critical link connecting periodontitis and systemic diseases.

## 4 Crosstalk between ferroptosis and cuproptosis in periodontitis

**Figure 1:**
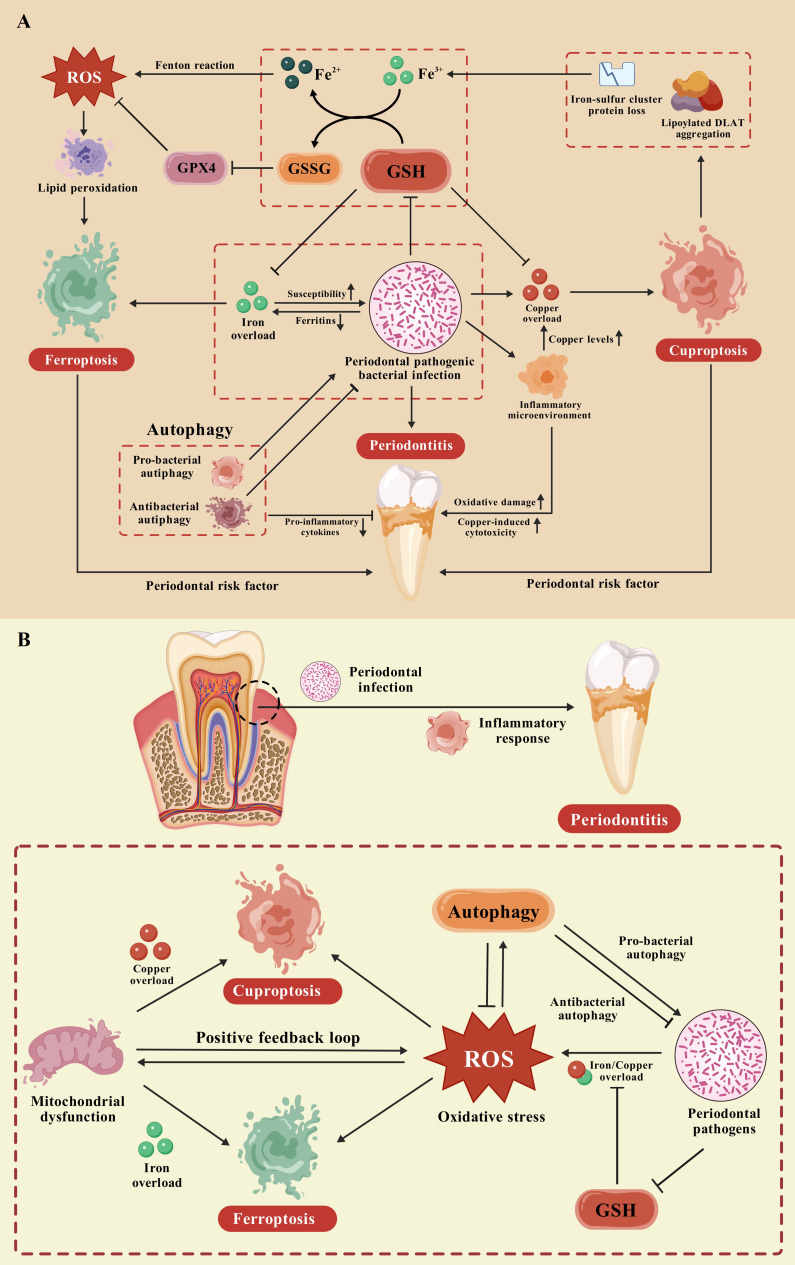
The potential crosstalk between ferroptosis and cuproptosis in periodontitis. **(A)** As possible periodontal risk factors, ferroptosis and cuproptosis are regarded as positively associated with the incidence and severity of periodontitis. A positive feedback loop exists between periodontal pathogens and iron concentration during periodontitis, ultimately triggering ferroptosis in the periodontium. The immunomodulatory responses to periodontal infection affect copper homeostasis in the periodontium, potentially resulting in the occurrence of cuproptosis and periodontitis. The increased inflammatory microenvironment may exacerbate copper toxicity and oxidative damage to periodontal tissue through further elevating copper levels. Notably, a self-accelerating cycle of ferroptosis and cuproptosis with robust proinflammatory potential may arise because of GSH consumption under attack by periodontal pathogens. Besides, autophagy exhibits both protective and pathological effects through pro-bacterial and antibacterial autophagy, ultimately regulating both ferroptosis and cuproptosis during periodontal inflammation. **(B)** The GSH pathway and autophagy pathway are closely related to alterations in oxidative stress levels during periodontitis. A homeostatic imbalance of copper and iron mediated by periodontal pathogens may contribute significantly to elevated periodontal oxidative stress. This phenomenon represents a unifying mechanism that elucidates the intricate interconnection between ferroptosis and cuproptosis in periodontitis.

Iron and copper are crucial metal elements involved in human physiological processes, such as cellular metabolism, oxygen transport, DNA and RNA synthesis, signaling transduction, and enzyme catalysis. A homeostatic imbalance of iron or copper plays a significant role in the pathogenesis and exacerbation of multiple diseases. Notably, increased intracellular copper levels may be important regulators of ferroptosis. Elesclomol-induced copper retention within mitochondria promoted oxidative stress and consequent ferroptosis in colorectal cancer cells via copper-transporting ATPase 1 (ATP7A) degradation and ROS accumulation (72). Copper-mediated GPX4 degradation drove autophagy-dependent ferroptosis via direct interaction of copper with GPX4 cysteines (Cys 107 and Cys 148) (73). Furthermore, consistent with the cuproptosis mechanism, blocking iron−sulfur cluster biogenesis could decrease mitochondrial lipoylation in brown adipose tissue, suggesting a regulatory role of iron in cuproptosis (74). Accumulating evidence has illuminated the mutual connections between ferroptosis and cuproptosis in various diseases (75–81). However, their interaction in periodontitis needs further clarification. This section describes the possible relevance of ferroptosis and cuproptosis in periodontitis pathogenesis (**Figure 1A**).

### 4.1 GSH pathway

Copper/iron chelator GSH may be a central signal mediator of both ferroptosis and cuproptosis, albeit with distinct roles in these processes. In ferroptosis, GSH functions as a cofactor for GPX4 activation to mitigate lipid peroxidation and ROS accumulation, whereas it serves as a copper chaperone to inhibit lipoylated protein aggregation in the TCA cycle in cuproptosis (13, 14). GSH synthesis suppresses both ferroptosis and cuproptosis, suggesting a potential unifying mechanism that clarifies their interconnection (13, 82, 83). Conversely, SLC7A11 blockade boosted the susceptibility of hepatocellular carcinoma cells to disulfiram/copper treatment, inducing both ferroptosis and cuproptosis via GSH depletion (84). More recent evidence also suggests that concurrent induction of these processes via GSH depletion may be a promising cancer treatment (85–87). Ferroptosis activators sorafenib and erastin could promote cuproptosis in primary liver cancer cells through elevating copper-dependent lipoylated protein aggregation via inhibiting GSH synthesis and mitochondria-dependent ferredoxin 1 (FDX1) protein degradation (88). Similar results were obtained in myelodysplastic syndromes cell lines (89).

GSH, a common biomarker of oxidative stress, may act as the intersection of ferroptosis and cuproptosis during periodontitis (26, 45, 90–92). Nevertheless, their crosstalk has not been established in periodontitis. We hypothesize that a self-accelerating cycle of ferroptosis and cuproptosis with robust proinflammatory potential may arise from GSH consumption under periodontal pathogen attack. Pathogen-stimulated iron overload induces ferroptosis through GSH depletion and mitochondrial dysfunction, resulting in excess intracellular copper and subsequent cuproptosis in the periodontium. Cuproptosis initiates lipoylated protein aggregation, iron–sulfur cluster protein loss, and ultimately the release of free Fe^3+^. The reaction between Fe^3+^ and GSH could further deactivates the GSH/GPX4 pathway through the transformation of GSH into oxidative GSH (GSSG) and provides abundant Fe^2+^ for the Fenton reaction, which accelerates ferroptosis in periodontal tissue. Consequently, targeting GSH may represent a novel approach to periodontitis treatment through simultaneous inhibition of ferroptosis and cuproptosis in periodontal cells. However, whether GSH pathway is involved in other PCD pathways in periodontitis and which pathway dominates periodontitis deserve further study.

### 4.2 Autophagy pathway

Autophagy is a complex cellular process contributing to the phagocytosis and degradation of various substrates, which maintains cellular homeostasis under normal physiological conditions and evokes autophagic cell death under pathological circumstances. Ferroptosis and cuproptosis are usually accompanied by autophagy activation, suggesting its role as a common hub for these processes. Multiple studies have demonstrated that selective autophagy plays a crucial role in the initiation of ferroptosis. Autophagy could act as a catalyst for ferroptosis to amplify iron-dependent lipid peroxidation and ROS accumulation through degradation of anti-ferroptotic factors, such as ferritin, GPX4, lipid droplets, and cadherin 2 (73, 93–99). The precise regulatory mechanism linking cuproptosis and autophagy remains incompletely understood. However, disruption in copper homeostasis can regulate the autophagy process through multiple pathways, triggering either a protective response or autophagic cell death based on stimulus strength and substrate properties (100–105). Moreover, cuproptosis improved chemosensitivity to docetaxel in prostate cancer cell lines by hindering autophagy via the dihydrolipoamide S-acetyltransferase (DLAT)/mammalian target of rapamycin (mTOR) pathway (106). The cuproptosis key gene ferredoxin-1 (FDX1) and its related genes were positively correlated with the expression of autophagy marker genes via recent bioinformatics analysis. Additionally, FDX1-mediated blockade of mitophagy also indicated a possible interplay between FDX1-mediated cuproptosis and autophagy (107, 108).

Existing data support the importance of autophagy as a bidirectional regulator of periodontitis pathogenesis, including cellular protection against apoptosis, enhancement of angiogenesis in periodontal tissues, promotion of other types of PCD, and regulation of alveolar bone homeostasis (109, 110). IL-17A-mediated iron metabolism prompted ferritin expression in osteoblasts, eventually bolstering osteogenic differentiation and alveolar bone repair via autophagy activation in murine periodontitis models. In addition, LPS-induced cuproptosis impeded autophagosome biogenesis and mitophagy in macrophages during periodontitis (66). Therefore, autophagy plays a significant role in both ferroptosis and cuproptosis, potentially serving as a central hub linking them in periodontal inflammation.

Autophagy exhibits both protective and pathological effects in periodontitis, which may constitute a significant factor contributing to the intricate influence of autophagy on ferroptosis and cuproptosis. This phenomenon is likely attributable to different responses of autophagy to bacterial infections, depending on the types of infected cells and periodontal pathogens (111). Autophagy can not only prevent periodontal infection and production of pro-inflammatory cytokines but also establish a unique microenvironment conducive to the replication and immune evasion of periodontal pathogens. Furthermore, excessive accumulation of iron and copper induced by periodontal pathogens could damage the antioxidant system in the periodontium, ultimately resulting in ferroptosis and cuproptosis during periodontitis. Thus, diverse responses of autophagy to periodontal infection may exert multifaceted effects on ferroptosis and cuproptosis through modulating the oxidative stress in periodontitis. Nevertheless, the specific regulatory mechanisms by which autophagy influences ferroptosis and cuproptosis in periodontitis remain elusive. Moreover, the precise relationship between ROS and autophagy initiation in periodontitis warrants further exploration for a comprehensive understanding of the association between ferroptosis and cuproptosis.

### 4.3 The potential unifying mechanism linking ferroptosis and cuproptosis in periodontitis

The GSH pathway and autophagy pathway are closely associated with alterations in oxidative stress levels during periodontitis. Oxidative stress can induce and interact with autophagy, whereas autophagy mitigates oxidative stress and confers cellular protection against oxidative damage in the periodontium. GSH functions as a critical antioxidant in the cellular defense against oxidative stress and is converted to GSSG through the action of GSH peroxidase in response to oxidative stress. A reduced GSH/GSSG ratio typically indicates the presence of oxidative stress and is widely recognized as a significant biomarker for the oxidative damage associated with periodontitis. In addition, increasing evidence suggests that mitochondrial dysfunction is implicated in the onset and progression of periodontitis. Fluctuations in mitochondrial function represent critical signaling events and serve as direct indicators of cellular responses to periodontal pathogen infection. Diverse manifestations of mitochondrial dysfunction, including oxidative stress, mitophagy, mitochondria-mediated apoptosis, and metabolic disorders, are commonly observed in periodontal disease (112–117). Interestingly, oxidative stress frequently drives mitochondrial dysfunction, which in turn aggravates oxidative stress through ROS overgeneration (118). Mitochondrial iron and copper are widely recognized as critical regulators of mitochondrial electron transport chain function and various other mitochondrial processes. Disruption of mitochondrial metal homeostasis can result in excessive ROS generation and induce ferroptosis and cuproptosis (119–122). Therefore, increased oxidative stress is likely to be a key factor in the shared pathophysiological mechanisms of ferroptosis and cuproptosis in periodontitis.

As previously discussed, the possibility that a positive feedback loop exists between periodontal infection and iron levels has been raised. Periodontal pathogens may trigger iron overload by degrading iron-binding proteins and destroy the antioxidant system in periodontal tissue, whereas increased iron concentrations further enhance the vulnerability to infection by periodontal pathogens and aggravate oxidative damage. Similarly, periodontal infection facilitates copper overload in the periodontal microenvironment to ensure an adequate supply of essential cuproenzymes, thereby intensifying periodontal oxidative damage and exacerbating the local inflammatory milieu. Additionally, extensive crosstalk occurs between copper and iron in various physiological and pathological processes; consequently, either iron imbalance or copper imbalance may simultaneously modulate both ferroptosis and cuproptosis through copper−iron interactions (72–74, 123–125). We infer that a homeostatic imbalance of copper and iron, which is mediated by periodontal pathogens, may constitute a significant factor contributing to elevated periodontal oxidative stress. This phenomenon potentially represents a unifying mechanism that elucidates the intricate interconnection between ferroptosis and cuproptosis in periodontitis (**Figure 1B**).

Antioxidant and antibacterial therapies are expected to become effective treatment options against ferroptosis and cuproptosis in periodontitis. Several natural antioxidant agents have been demonstrated to be promising approaches against oxidative damage in the context of periodontitis. Quercetin has been shown to decrease oxidative damage in periodontal ligament cells through the activation of NRF2 signaling while also reducing alveolar bone loss in murine models of experimental periodontitis (126). Silibinin has demonstrated significant anti-inflammatory and antioxidative effects against periodontitis both *in vitro* and *in vivo* by downregulating the expression of NF-κB and NLRP3 while upregulating NRF2 expression (127). Similarly, resveratrol can prevent alveolar bone loss in an experimental rat model of periodontitis by ameliorating the production of circulating ROS via the NRF2/HO-1 axis (128). The local administration of curcumin gel also exerts an antioxidant effect on attenuating ligature-induced periodontitis in diabetic rats (129). In addition, novel treatment alternatives against periodontal pathogens, such as zinc materials, probiotics, N-chlorotaurine and antimicrobial photodynamic therapy, are being explored (130–134).

## 5 Conclusions and perspective

In conclusion, these findings revealed the involvement of ferroptosis and cuproptosis in the pathogenesis of periodontitis. Although the cause-and-effect relationship between ferroptosis and cuproptosis remains unclear, we propose several potential associations of these two forms of PCD in periodontitis, including the GSH pathway and the autophagy pathway. Elevated oxidative stress may constitute a unifying mechanism underlying the complex interrelationship between ferroptosis and cuproptosis in periodontitis. Antioxidative and antibacterial therapies may serve as efficacious treatment modalities for periodontal damage through inhibition of ferroptosis and cuproptosis.

Future research is imperative to address the following issues. The biomarkers of ferroptosis and cuproptosis in periodontal disease, as well as their precise relationships with other forms of PCD in periodontitis pathogenesis, remain poorly understood. The overlapping characteristic markers (*e.g.*, iron, copper, p53, ROS, and inflammatory mediators) underscore the potential crosstalk among different PCD forms during periodontitis. Targeting shared nodes may yield synergistic therapeutic effects, offering novel combinatorial regimens to halt periodontitis progression. Furthermore, the disparity between animals and humans has contributed to the perception that the effects of ferroptosis inhibitors or cuproptosis inhibitors on periodontitis observed in animals may not be reliably replicated in humans. The potential effects of inhibiting ferroptosis and cuproptosis in periodontitis on the proliferation and dissemination of periodontal pathogenic bacteria necessitate further investigation.

Elucidating the roles of ferroptosis and cuproptosis in periodontitis may yield novel insights for future investigations to explore the specific pathogenic mechanisms underlying periodontitis.
